# Special Issue “Biosynthesis and Application of Natural Compound”

**DOI:** 10.3390/ijms27031226

**Published:** 2026-01-26

**Authors:** Olga A. Luzina, Konstantin P. Volcho

**Affiliations:** N. N. Vorozhtsov Novosibirsk Institute of Organic Chemistry, Siberian Branch of the Russian Academy of Sciences, 9, Lavrentieva Ave., Novosibirsk 630090, Russia; luzina@nioch.nsc.ru

## 1. Introduction

Natural compounds are widely used in medical practice in their native form [1,2,3,4,5] and also play a key role in the development of new drugs [6,7,8,9,10,11]. The transition from natural raw materials to a medicinal product includes a combination of several important stages. The first step is often the study of the biological activity of extracts, which can either result in the development of a botanical drug or become the basis for the identification of individual compounds responsible for the biological effect. In the successful discovery of such biologically active compounds, the next important issue to address is their production in the large quantities required for in-depth testing. This is often a complex problem, as target compounds may be present in extracts in trace amounts, in a difficult-to-separate mixture with structurally similar analogs. Possible solutions include the complete synthesis of target compounds or targeting biosynthetic pathways to increase their content in extracts, even eliminating the need for whole-organism cultivation. Finally, readily available natural compounds can serve as the basis for further chemical transformations, enabling either enhancement of their native biological activity or the creation of derivatives with new biological properties. This Special Issue, “Biosynthesis and Application of Natural Compound,” brings together articles devoted to modern research in this area, aiming to address these issues.

## 2. An Overview of Published Articles

Society’s need for biologically active substances of natural origin, including for the development of prophylactic and therapeutic drugs, is reflected in studies investigating the biological activity of phytoextracts and isolating individual compounds responsible for their specific activity.

One of the inherent qualities of natural substances is their adjuvant properties, which could enhance the activity of drugs to which high levels of resistance have been developed. A striking example of this type of activity is opeisan, as demonstrated by Dofini Magnini R. et al. (contribution 1). The authors demonstrated that a hydroethanolic extract of *Acacia senegal* leaves can increase the permeability of the outer membrane of Gram-negative bacteria *Escherichia coli* and *Klebsiella aerogenes* and exhibits low toxicity. Fractionation of the extract and evaluation of the effects of the fractions, both individually and in combination with chloramphenicol, on resistant bacterial strains allowed the authors to identify the components responsible for this effect, which are derivatives of the macrocyclic alkaloid budmunchiamine.

Kim H. et al. (contribution 2) used an approach based on culturing *Hibiscus sabdariffa* plant cells obtained from leaf tissue. This led to minimization of the consumption of plant materials and isolation of the dipeptide Gly-Pro (GP), which was found to be capable of suppressing collagen production induced by TGF-β1. The peptide inhibited the phosphorylation of Smad2/3 and reduced the expression of ATF4, which is upregulated by TGF-β1. Notably, GP also decreased the expression of enzymes involved in the serine/glycine biosynthesis and glucose metabolism pathways, such as PHGDH, PSAT1, PSPH, SHMT2, and SLC2A1. The authors’ studies demonstrate the potent antifibrotic effect of the dipeptide Gly-Pro.

Biotechnological approaches were also used by Kowalczyk T. et al. (contribution 3) in their study, which examined extracts of the hairy roots of *Senna obtusifolia*, including roots with overexpression of the gene-encoding squalene synthase 1. Both extracts demonstrated anti-inflammatory and wound-healing activity, but the extract of transgenic hairy roots had stronger biological properties, which may result from its higher content of bioactive secondary metabolites.

The importance of understanding the biosynthetic pathways used for producing biologically active natural compounds in large quantities is reflected in the works of Chen X. (contribution 4) and Hua C. (contribution 5), who demonstrate that transcriptome studies of the traditional botanical remedies *Ligusticum chuanxiong* and *Physalis angulata* reveal enzymes (and signaling pathways) involved in the biosynthesis of the pharmacologically significant substances butylphthalide and physalin, respectively. Moreover, the authors used different approaches to identify the genes involved in the biosynthesis process. Chen et al. identified the plant organ in which butylphthalide accumulates and, by comparing transcriptome data from various *L. chuanxiong* tissues, identified four rhizome-specific genes, denoted 2-oxoglutarate-dependent dioxygenases (2-OGDs). Hua et al. developed a virus-induced gene-silencing (VIGS) system for *P. angulata*. Conclusions about the involvement of P450 candidates in the physalin biosynthetic pathway were drawn from data on the reduction in the transcripts of the four P450 candidates by VIGS, which correlated with decreased levels of physalin-class compounds in the *P. angulata* leaves.

A study by Cho W.K. et al. (contribution 6) found that aqueous extracts obtained from roselle plants (HSPE) and callus cells (HSCE) had different compositions. A transcriptome analysis of treated human skin cells explained the observed differences in the biological activity of wild-type and callus cultures of Roselle (*Hibiscus sabdariffa* L.) (HSPE and HSCE, respectively), with HSPE having greater effects on human skin cells. Up-regulated genes by HSPE show function in angiogenesis, the oxidation reduction process, and glycolysis, while up-regulated genes by HSCE encode ribosome proteins and IFI6, functioning in the healing of radiation-injured skin cells. Callus culture was noted to have a strong anti-melanogenic effect and perform skin barrier functions and antioxidant activity.

Using traditional biosynthesis with lipase, Capecchi E. et al. (contribution 7) obtained novel derivatives of ascorbic acid 3-*O*-ethyl-*L*-ascorbyl-6-ferulate and 3-*O*-ethyl-*L*-ascorbyl-6-palmitate. The application of lignin nanoparticles for its encapsulation improved stability against temperature- and pH-dependent degradation and antioxidant activity.

Ye S. et al. (contribution 8) demonstrated that, by activating a specific gene cluster in *Streptomyces* genomes, it is possible to obtain/isolate new, previously undescribed metabolites, which the authors named ahbamycins (AHBs). These constitute a new family of metabolites derived from 3-amino-4-hydroxybenzoate (3,4-AHBA), known for their antibiotic and antitumor activity. The authors’ approach opens the possibility of obtaining new compounds through combinatorial biosynthesis.

Łój et al. (contribution 9) used the fact that entomopathogenic fungi are known to catalyze O-glycosylation reactions to target the biotransformation of the natural compound xanthohumol into glycosides to increase solubility. As a result of the experiments, five glycosides were obtained, including one that has never been described in the literature so far. Interestingly, in addition to the expected glycosylation reactions, the authors showed that the tested fungi also catalyzed chalcone–flavanone cyclization reactions, which demonstrate chalcone isomerase-like activity, an enzyme typically found in plants.

Total synthesis is one of the classic approaches to obtaining natural compounds. Nam S. et al. (contribution 10) described an asymmetric total synthesis of a series of natural compounds (*S*)-daphneolone and (*S*)-dihydroyashabushiketol, as well as the formal synthesis of (3*S*,5*S*)-yashabushidiol B, using readily available (3*S*)-hydroxy-5-phenylpentanoic acid. The synthetic route developed by the authors offers a versatile approach to the synthesis of linear diarylpentanoids and diarylheptanoids containing a β-hydroxyketone or 1,3-diol functional group as a structural motif.

Chemical modifications of natural compounds remain a highly sought-after approach for synthesizing new pharmacologically active agents with either enhanced target activity or a new spectrum of activity. Jeong G.H. et al. (contribution 11) thermolyzed the isoflavonoid rotenone and demonstrated that the thermolysis products exhibited greater anti-inflammatory activity than rotenone itself. New dihydrobetulin derivatives obtained by Tolmacheva I. et al. (contribution 12) by the selectively modified A ring of the natural triterpenoid exhibit higher cytotoxic activity than their original compound. The antitumor activity of the new compounds is presumably due to activation of the mitochondrial pathway, mediated by reactive oxygen species. A similar trend was described by Shih Y.-H. et al. (contribution 13), who synthesized new caffeic acid amides and studied their anticancer properties and mechanisms of action on oral squamous cell carcinoma cell lines SAS and OECM-1. The results showed that the synthesized compounds induce cancer cell death by activating autophagic signaling, which correlates with changes in cellular oxidative stress.

Substances with new properties were obtained by Bogdanov A.V. et al. (contribution 14) and Samorodov A.V. et al. (contribution 15). In the first study, water-soluble quaternary ammonium acylhydrazones based on catecholaldehyde were synthesized. These derivatives exhibit antioxidant and antibacterial properties against Gram-positive bacteria, including methicillin-resistant *Staphylococcus aureus* strains. The antimicrobial effect of the most active acylhydrazones was shown to be associated with the destruction of the bacterial cell wall. Depending on their structure, hydrazones exhibit anti- or pro-aggregation properties. Hydrazones of isatin, an oxidation product of the natural indigo dye, containing a phosphorus-containing fragment, obtained in the study by Samorodov, A.V. et al., were more effective than acetylsalicylic acid under the conditions of the tissue factor (TF)-activated thromboelastography (TEG) model, an ex vivo thrombosis model. Both ammonium acylhydrazones based on catecholaldehyde and isatin hydrazones can serve as a basis for the creation of antiplatelet drugs with broad antithrombotic potential.

The work by Aleksandrova Y. (contribution 16) shows that hydroxamic acid containing monoterpenoid-derived para-menthene backbone exhibits potent inhibitory activity against HDAC6 and demonstrated significant ability to suppress the aggregation of pathological β-amyloid peptide, a hallmark feature of Alzheimer’s disease. They also demonstrated neuroprotective effects of the derivative on in vivo models of the disease using 5×FAD transgenic mice.

Podturkina A.V. et al. (contribution 17) were the first to study the inhibitory activity of derivatives of the natural monoterpenoid (−)-verbenone in relation to the enzymes MAO-A and MAO-B. It was found that some derivatives (antiparkinsonian agent Prottremine and its 9-*N*- and *S*-derivatives) inhibit the enzyme MAO-B, which may contribute to the antiparkinsonian activity of these compounds. Another monoterpene motif, bornyl, has proven useful in the development of agents with significant hypoglycemic activity in the oral glucose tolerance test with CD-1 mice (Kuranov S.O. et al.; contribution 17). The compounds were confirmed to have targeted activity against free fatty acid receptor-1 (FFAR1).

## 3. Conclusions

The works presented in this issue reflect modern achievements in the field of chemistry of natural compounds, including studying the biological activity of extracts, individual natural compounds, and their derivatives, as well as targeting biosynthetic pathways to increase the content of selected compounds in extracts. Further directions, besides the mentioned ones, could include the investigation of the ADMET properties of natural products and their derivatives and the optimization of these properties through the development of effective delivery systems. Moreover, adjuvant properties of natural compounds and their derivatives in various therapeutics areas also deserve more attention.
